# Co-Microencapsulation of Flavonoids from Yellow Onion Skins and Lactic Acid Bacteria Lead to Multifunctional Ingredient for Nutraceutical and Pharmaceutics Applications

**DOI:** 10.3390/pharmaceutics12111053

**Published:** 2020-11-04

**Authors:** Ștefania Adelina Milea, Mihaela Aida Vasile, Oana Crăciunescu, Ana-Maria Prelipcean, Gabriela Elena Bahrim, Gabriela Râpeanu, Anca Oancea, Nicoleta Stănciuc

**Affiliations:** 1 Faculty of Food Science and Engineering, Dunarea de Jos University of Galati, 111 Domnească Street, 800201 Galați, Romania; Adelina.Milea@ugal.ro (Ș.A.M.); Mihaela.Vasile@ugal.ro (M.A.V.); Gabriela.Bahrim@ugal.ro (G.E.B.); Gabriela.Rapeanu@ugal.ro (G.R.); 2 National Institute of Research and & Development for Biological Sciences, 296 Splaiul Independentei, 060031 Bucharest, Romania; oana.craciunescu@incdsb.ro (O.C.); anamaria.prelipcean@incdsb.ro (A.-M.P.); anca.oancea@incdsb.ro (A.O.)

**Keywords:** yellow onion skins, flavonoids, lactic acid bacteria, co-microencapsulation, antioxidant activity, metabolic syndrome, cytotoxicity, functionalization

## Abstract

In this study, flavonoids extracted from yellow onion skins and *Lactobacillus casei* were encapsulated in a combination of whey protein isolate, inulin and maltodextrin with an encapsulation efficiency of 84.82 ± 0.72% for flavonoids and 72.49 ± 0.11% for lactic acid bacteria. The obtained powder showed a flavonoid content of 89.49 ± 4.12 mg quercetin equivalents/g dry weight (DW) and an antioxidant activity of 39.27 ± 0.45 mM Trolox/g DW. The powder presented a significant antidiabetic and anti-inflammatory potential, with an inhibitory effect on α-amylase, lipase and lipoxygenase of 76.40 ± 2.30%, 82.58 ± 3.36% and 49.01 ± 0.62%, respectively. The results obtained for in vitro digestion showed that the coating materials have a protective effect on the flavonoids release. Cytotoxicity results indicated that the powder was cytocompatible up to a concentration of 500 μg/mL. The functional potential of the powder was tested by adding in a selected food matrix, highlighting a good stability of the phytochemicals, whereas an increase with 1 log cell forming unit (CFU)/g DW was observed after 21 days of storage. The obtained results are promising in the valorization of natural antioxidants in combination with lactic acid bacteria in order to develop multifunctional ingredients with value-added for food and pharmaceutics applications.

## 1. Introduction

Numerous plant components have been associated with the prevention and management of many chronic diseases. The skin or outer scales of yellow onion bulbs contain high amounts of bioactive compounds, such as phenolic and flavonoids compounds, mostly in their glycosides form. Flavonoids are natural pigments responsible for the coloring in fruits and vegetables [1]. They are known for their biological effects, including anti-oxidative, anti-carcinogenic, anti-inflammatory and anti-mutagenic properties. Quercetin is probably one of the most studied natural antioxidants, due to its potential to prevent and/or slow the formation of free radicals or reactive species resulted via oxidative stress, which, nowadays, is one of the most investigated properties of polyphenols, from the perspective of developing new, natural alternatives for cancers prevention and treatment [1]. In addition, direct correlation between oxidative stress and insulin resistance has been reported. Therefore, the flavonoids extracted from yellow onion skins may exhibit important inhibitory activity against targeted enzymes associated with metabolic syndrome, such as α-amylase and lipase and against pro-inflammatory enzymes, such as lipoxygenase [2]. Metabolic syndrome is known as a group of conditions that occur at the same time, with serious consequences, such as increased risk of heart disease, stroke and type 2 diabetes. The effects of these consequences are increased blood pressure and blood sugar, obesity, and abnormal cholesterol or triglyceride levels. For these reasons, quercetin is suggested to be involved in remarkable beneficial health effects. However, due to their molecular and structural particularities, this class of compounds shows high sensitivity to external environmental conditions, such as light, heat treatments, pH changes, the presence of oxygen, ions, proteins, etc. [3]. Therefore, their effectiveness in promoting health benefits is highly dependent on preserving the stability, bioactivity, and bioavailability of the active ingredients [4].

The health benefits of probiotics have been recognized as key health promoters. Probiotics are known for their ability to contribute to the regulatory processes. Therefore, probiotics stimulate, modulate and regulate the host’s immune response, modulate the gastrointestinal hormone release and regulate brain behavior [5]. They play a significant role in inducing intestinal angiogenesis, and regulate acute and chronic inflammation in intestinal mucosal tissue [5]. However, lactic acid bacteria (LAB) have specific growth conditions, lacking the ability to survive extreme temperature and pH conditions [6].

To overcome the poor processing and bioavailability of polyphenols and probiotics, various microencapsulated delivery systems have been developed, therefore improving the taste, providing protection from processing conditions and improving absorption through the intestinal tract. Encapsulation is a technique aimed to entrap flavonoids and probiotic bacteria in gastro-resistant polymers and to carry them directly to the intestine [7]. Microencapsulation is useful to develop new food ingredients in order to improve the functional applications of the flavonoids and probiotics.

In our study, flavonoids and probiotics were co-microencapsulated by freeze-drying using whey protein isolate, maltodextrin and inulin with the aim to assess a profile of co-microencapsulated flavonoids from yellow onion skins extract and LAB and to evaluate the efficacy of phenolic against free radicals and enzymes relevant in hyperglycemia, dyslipidemia, oxidative stress and the inflammatory process related with metabolic syndrome. In order to evaluate the antioxidant activity, the powder was analyzed for total polyphenol (TPC) and total flavonoid (TFC) contents. In addition, to predict the biological activity, the lipase, α-amylase, lipoxygenase inhibitory effect of the powder was evaluated. The in vitro biocompatibility was assessed on fibroblast cells by testing the cell viability, cell proliferation and cell morphology in the presence of co-microencapsulated powder. The functional value of the powder was tested by adding it in a suitable food matrix, which was further tested for global phytochemical compound stability in relation to the antioxidant activity and the viability of the bacterial cells during storage. These results bring certain advantages in terms of exploiting the beneficial effects of both flavonoids and probiotics, for developing new valuable ingredients with improved functional properties.

## 2. Materials and Methods

### 2.1. Materials

Yellow onion (*Allium cepa*) was purchased from the local market (Galați, Romania) in August 2019. The onion samples were visually inspected, washed, and the skins were separated manually and washed again with distilled water. The skins were subjected to moderate drying at 40 °C for 2 h, followed by storage at 4 °C until further analysis. Whey proteins isolate (WPI) (protein content of 95%) was purchased from Fonterra (Auckland, New Zeeland). Inulin (I) from chicory and maltodextrin (MD) (DE dextrose equivalent 16.5–19.5) were purchased from Sigma-Aldrich Corp. (St. Louis, MO, USA). The reagents used to determine the total phenolic compounds (TPC), total flavonoids content (TFC), 2,2′-azino-bis(3-ethylbenzothiazoline-6-sulfonic acid (ABTS), 2,2-diphenyl-1-picrylhydrazyl (DPPH), 6-hydroxy-2,5,7,8-tetramethylchroman 2-carboxylic acid (Trolox) were purchased from Sigma-Aldrich Corp. (St. Louis, MO, USA). The commercial culture *Lactobacillus casei* ssp. *paracasei* (*L. casei* 431^®^) was provided by Chr. Hansen (Hoersholm, Denmark). MRS agar (de Man, Rogosa and Sharpe) from Merck (Eppelheim, Germany) was used for evaluation of the viability of the *L. casei* 431^®^.

### 2.2. Methods

#### 2.2.1. Flavonoid Extraction from Yellow Onion Skins

For the flavonoid extraction from yellow onion skins, we selected a combined extraction method, based on our previous results [4], by using the solid-liquid ethanolic method combined with ultrasound assisted extraction. Therefore, an amount of 50 g of ground onion skin was weighted and mixed with 450 mL of 70% acidified ethanol solution. The extraction was performed by ultrasonication for 30 min at 40 °C, followed by centrifugation at 6000× *g* for 15 min and 4 °C (MRC Scientific 193 Instruments, Holon, Israel). In order to intensify the extraction yield, the supernatant was collected, and the extraction was repeated 3 times. After the extraction, the collected supernatants were concentrated to dryness under reduced pressure at 40 °C (AVC 2-18, Christ, UK).

#### 2.2.2. Microencapsulation of Flavonoids and LAB

An amount of ~7 g of extract was dissolved in 500 mL of ultrapure water, followed by extracting global phytochemical characterization and co-microencapsulation. The microencapsulation of flavonoids from yellow onion skins by freeze-drying was performed by adding WPI, MD and I in a ratio of 2:1:1 (*w*:*w*:*w*). The mixture was stirred on a magnetic stirrer for 20 h until hydration of the biopolymeric materials. Afterwards, the solution was sterilized for 1 h using a UV lamp and inoculated with 2 g of *L. casei* 431^®^ lyophilized culture, followed by freeze-drying (CHRIST Alpha 1–4 LD plus, Osterode am Harz Germany) at −42 °C under a pressure of 10 Pa for 48 h.

#### 2.2.3. Determination of Selected Phytochemical Profile of the Extract and Co-Microencapsulated Powder

In order to determine the phytochemical profile of the extract and co-microencapsulated powder, the methods previously described by Oancea et al. [8] were used. The extract and the powder were characterized in terms of TFC, TPC and antioxidant activity by using the aluminum chloride method, Folin-Ciocâlteu, DPPH and ABTS radicals scavenging method, respectively. The encapsulation efficiency of the powder was calculated by comparing the values for surface flavonoids content (SFC) and total flavonoids content (TFC), as described by Saénz et al. [9].

#### 2.2.4. Encapsulation Efficiency and Viability of LAB

The procedure described by Colín-Cruz et al. [10] was used for LAB co-microencapsulation efficiency estimation. The quantification of the viable bacteria was performed by pour plate technique. The percentage (%) of efficiency was determined according the following equation:

(1) EE%=NN0×100

where *N* is the number of viable cells (cell forming units- CFU/g DW) in the powder, and *N*_0_ is the number of viable cells in the solution (CFU/g DW) before the freeze-drying process.

To determine the viable cell counts of LAB, the pour plate technique was used. After serial dilutions of the dissociated cells from the samples, the number of surviving cells was counted by plate culture on MRS agar in duplicate and aerobically incubated at 37 °C for 48 h. The count was expressed as colony forming units per g dry weight (DW) [11].

#### 2.2.5. Determination of Physical Properties of the Microencapsulated Powder

##### Powder Solubility

The solubility of co-microencapsulated powder was measured by using a method described by Hussain et al. [12]. An amount of 0.5 g of powder was weighted and mixed with 50 mL of distilled water and stirred with a magnetic stirrer for 20 min. The solution was then centrifuged at 5100× *g* at 4 °C, for 10 min and the supernatant was collected. A volume of 25 mL of solution was transferred to a Petri Dish and oven dried at 105 °C for 5 h. The solubility was calculated as a weight difference and represented as a percentage (%). The experiments were performed in triplicates.

##### Powder Hygroscopicity

An amount of 0.5 g of powder was placed in a desiccator with saturated NaCl solution (75%), at 26 °C. The hygroscopicity was calculated after 1 week by weighting the sample and was expressed in the term of percentage (%) [12].

#### 2.2.6. In Vitro Assays

Inhibitory activity of enzymes related to metabolic syndrome and pro-inflammatory processes.

##### α-Amylase Inhibition

A volume of 100 μL of powder solutions (10 mg/mL in 0.1 M PBS, pH 6.9) was added to 100 μL of 13-U/mL α-amylase solution (1 mg/mL in 0.1 MPBS, pH 6.9). The mixture was allowed to react for 10 min at 37 °C, then 100 μL of 1% soluble starch solution in PBS, previously boiled for 5 min, was added to each tube and incubated for another 10 min. Finally, a volume of 200 μL of dinitro salicylic acid reagent was added to each test tube, followed by heating at 100 °C for 5 min in a water bath. Further, the samples were diluted with 2 mL of distilled water and absorbance read at wavelength of 540 nm. Enzyme inhibition was calculated using Equation (2):

(2) $$\%\mathrm{Inhibition}=\frac{\left(A_{0}-A_{s}\right)}{A_{0}}\times 100$$

##### Lipase Inhibition

Lipase activity was assayed according to Costamagna et al. [2] by calculating the enzymatic hydrolysis of *p*-nitrophenyl palmitate to *p*-nitrophenol. Lipase (concentration 1.0 mg/mL) was firstly mixed with co-microencapsulated powder (concentration 10 mg/mL) and kept at 25 °C for 5 min. Then, 330 µL of substrate supplemented with Triton X-100 (0.6% *w*/*v*) and 0.15% Arabic gum was added to the mixture. The solution was incubated at 37 °C for 20 min and the inhibition was determined using the Equation (2). The absorbance was read at 400 nm.

##### Lipoxygenase Inhibition

Lipoxygenase activity was measured using a spectrophotometric method based on the enzymatic oxidation of linoleic acid to hydroxiperoxide, according to Costamagna et al. [2]. A volume of 50 µL of lipoxygenase solution (concentration 1 mg/mL in borate buffer pH 9.0) was mixed with 50 µL of powder solution (10 mg/mL) and pre-incubated on room temperature for 5 min. The substrate was then added in the mixture, incubated at 37 °C and the inhibition of hydroperoxide release was measured at 234 nm.

##### Determination of Bioavailability of Bioactives

The estimation for bioavailability of co-microencapsulated powder was determined by the method described by Oancea et al. [8]. The in vitro digestibility of the flavonoids from powder was performed by using a static model that simulates digestion in the stomach and intestine.

#### 2.2.7. In Vitro Cytotoxicity of the Co-Microencapsulated Powder

##### Cell Culture and Treatment

For cell culture and treatment, the mouse fibroblasts from NCTC clone L929 cell line (ECACC, Sigma-Aldrich, Darmstadt, Germany) were cultured in Minimum Essential Medium (MEM), which was supplemented with 10% (*v*/*v*) fetal calf serum (FCS), 2 mM L-glutamine and 1% (*v*/*v*) antibiotic mixture (penicillin–streptomycin–neomycin). The cells were cultured in a humidified atmosphere with 5% CO_2_, at 37 °C, until subconfluence. The powder was prepared in culture medium as stock solutions, at a starting concentration of 1 mg/mL, followed by incubation at 37 °C for 24 h, followed by filtering through 0.22 µm membrane (MilliporeSigma, Merck, Darmstadt, Germany).

The preparation of cell suspension was made by adding trypsin over the cells in order to detach them. The cells were introduced in 24-well microplates at a density of 4 × 10^4^ cells/mL and incubated at 37 °C, in humidified atmosphere with 5% CO_2_. After 24 h, the culture medium was replaced with fresh medium containing co-microencapsulated powder in a concentration ranging from 10 to 1000 μg/mL. The samples were then incubated at 37 °C, for 24 and 48 h, respectively. The control culture containing culture medium without powder was considered.

##### Cell Viability

Cell viability was determined by a spectrophometric method, using Neutral red (NR) as an indicator, as described by Gaspar et al. [13]. After 24 and 48 h of incubation, the culture medium was removed from each well and replaced with 50 μg/mL NR solution. The microplates containing mixture were incubated for 3 h at 37 °C. The cells were washed, and the dye was released by gentle shaking for 15 min using 1% (*v*/*v*) acetic acid solution in 50% (*v*/*v*) ethanol. The amount of removed dye was directly proportional to the number of viable cells. The optical density was measured at 540 nm on a Sunrise microplate reader (Tecan, Männedorf, Austria). The control culture was considered 100% viable and the results are expressed as the percentage relative to it.

##### Cell Morphology

Cell morphology after 48 h of cells incubation in the presence of samples was observed by light microscopy. Micrographs were acquired at an optical microscope Axio Observer D1 equipped with a digital camera (Carl Zeiss MicroImagining, Göttingen, Germany).

#### 2.2.8. Formulation of a Soft Cheese with Added Value

In order to support the multifunctional properties of bioactives and LAB from co-microencapsulated powder, the ingredient was tested for storage stability for 21 days at 4–6 °C, both for phytochemical profile and for cells viability of LAB. A cream cheese was selected due to its compositional particularities, allowing us to maintain and improve the stability of polyphenols and LAB. Two variants of value-added soft cheese were prepared. An amount of 50 g of cheese cream was weighed for each sample. For the first variant, the powder was added in a ratio of 1% (V1) and in a ratio of 2% (V2). The control sample was a cheese cream without microencapsulated powder addition (C). Samples were homogenized and allowed to stand for 1 h at room temperature to equilibrate them. The samples were homogenized then analyzed for phytochemical stability and cell viability over 21 days.

#### 2.2.9. Statistical Analyses

All analyses were performed in triplicate and data are reported as mean ± standard deviation (SD). In order to identify significant differences, experimental data were subjected to one-way analysis of variance (ANOVA) after running the normality and homoscedasticity tests. The Tukey method with a 95% confidence interval was employed for post-hoc analysis; *p* < 0.05 was considered to be statistically significant. The statistical analysis was carried out using Minitab 18 software.

## 3. Results and Discussions

### 3.1. Extract Characterization, Co-Microencapsulation and Global Phytochemical Characterization of the Powder

The extract and powder global phytochemical characterization are given in Table 1. The yellow onion skins extract showed a TFC of 229.14 ± 3.05 mg quercetin equivalents (QE)/g dry weight (DW), a TPC of 96.06 ± 2.70 mg gallic acid equivalents (GAE)/g DW and an antioxidant activity of 101.19 ± 0.53 mM Trolox/g DW.

The yellow onion skin extract showed a TFC of 229.14 ± 3.05 mg quercetin equivalents (QE)/g dry weight (DW), a TPC of 96.06 ± 2.70 mg gallic acid equivalents (GAE)/g DW and an antioxidant activity of 101.19 ± 0.53 mM Trolox/g DW (Table 1). In a previous study, Milea et al. [4] suggested lower concentrations of bioactive compounds (TFC of 97.28 ± 3.01 mg QE/g DW and TPC of 55.27 ± 2.47 mg GAE/g DW), but a higher antioxidant activity of 344.97 ± 2.68 mM Trolox/g DW. Singh et al. [14] extracted bioactive compounds from onions using 70% ethanol on ultrasound assisted extraction and obtained similar values for flavonoids of 212.3 ± 14.6 mg QE/g DW and a higher number of phenolic compounds (418.0 ± 34.4 mg GAE/g DW). These differences may be given by the phytochemical variability in raw material and the type of solvent combination used in the extraction.

One of the major objectives of this study is to improve the stability and bio-disponibility of the phenolic extracted from onion skins by co-microencapsulation, in order to obtain a multifunctional ingredient. It has been reported that one of the most important parameters evaluating the ability to include a bioactive compound in the encapsulant material is the encapsulation efficiency [3]. The combination of WPI, I and MD, as a coating material, led to an encapsulation efficiency of 84.82 ± 0.72%. Different encapsulation efficiency values were reported by Akdeniz et al. [15], varying between 55.64% and 89.15% for encapsulating phenolic from onion skins in different coating material combinations, with a maximum value found for maltodextrin:casein in a ratio of 6:4.

The microencapsulation efficiency of LAB in freeze-dried powder was 72.49 ± 0.11%. Gebara et al. [16] suggested that a comparison between data of encapsulation efficiency of LAB reported in the literature is complex, due to the wide range of microorganisms, encapsulation techniques and encapsulation materials used. Thus, our results are significantly higher than a microencapsulation efficiency of 36.90% for *Lactobacillus acidophilus* cells in alginate microcapsules obtained by internal emulsification/gelling [17] and of 38.00% for *Lactobacillus casei* and *Lactobacillus plantarum* [18] co-encapsulated with a selenium-enriched green tea in chitosan-coated alginate microcapsules obtained by extrusion.

The global phytochemical profile of the powder showed a content of TFC of 89.49 ± 4.12 mg QE/g DW and TPC of 34.17 ± 1.79 mg GAE/g DW, yielding a DPPH radical scavenging activity of 39.27 ± 0.45 mM Trolox/g DW, with an inhibition of 87.40 ± 0.95% (Table 1). Horincar et al. [19] used whey protein-chitosan and whey protein-maltodextrin-pectin as wall materials in different ratios and reported values for total flavonoid content of 5.84 ± 0.23 mg QE/g DW and 104.97 ± 5.02 mg QE/g DW, respectively, with an antioxidant activity of 175.93 ± 1.50 mM Trolox/g DW and 269.20 ± 3.59 mM Trolox/g DW, respectively. The difference in concentration of polyphenolics could be explained by the initial low concentration of polyphenolics from the extract and by a different core:wall material ratio used by other authors.

### 3.2. Solubility and Hygroscopicity of the Co-Microencapsulated Powder

Physical factors, such as hygroscopicity are indispensable for encapsulated products stability, storage and practical applications, whilst aqueous solubility is correlated with ability of powder products for reconstitution [20]. Various factors that determine the solubility of the microencapsulated powders could include their composition, particle size and final food matrix. Solubility is an important instant property for encapsulated flavonoids because it may be subjected to rehydration when used as a food ingredient [21].

In our study, co-microencapsulated powder exhibited a water solubility index of 65.07 ± 1.24%. This result may be attributed to the microencapsulation technique consisting in 20 h of time mixing and also to the complexity of the encapsulation material. The observed result can also be correlated to the particle size distribution. Fortunately, the final solubility depends mostly on the composition of the final food product, which would ultimately provide the better surface area’s availability for the hydration process. Syamaladevi et al. [21] studied the water solubility index for encapsulated raspberry powder and reported similar results varying from 61.7% to 70.1%. Hussain et al. [12] suggested higher aqueous solubility for some microencapsulated polyherbal formulas ranging from 84.06% to 92.31%.

Hygroscopicity yielded a value of 11.9 ± 0.70%. Different values ranging from 11.92% to 14.25% for freeze-dried samples encapsulated with gum Arabic, gelatin, and maltodextrin were reported by Hussain et al. [12]. It has been suggested that lyophilized products have lesser hygroscopic values [22]. In the case of freeze-dried products, lower values for hygroscopicity are generally attributed to the larger particle size, which leads to a higher percentage of uncovered surface area, and, as a consequence, a lower degree of water absorption. Nevertheless, hygroscopicity is a favorable property, considering the solubility of a powder during rehydration [20].

### 3.3. Flavonoids Stability in Gastrointestinal Simulated Conditions

**Figure 1:**
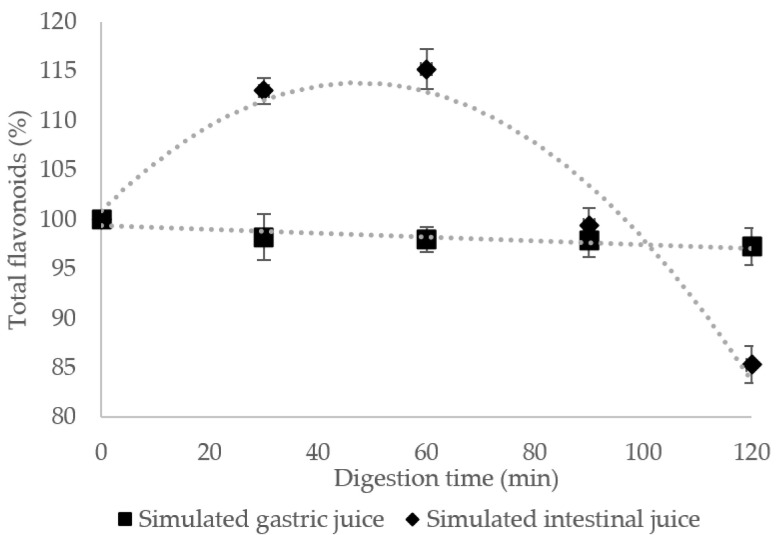
Total flavonoids release from the co-microencapsulated powder during in vitro digestion in simulated gastric and intestinal juices. Data shown are averages of triplicate samples. Error bars on the chart represent standard deviations.

It is often mentioned that microencapsulation is designed to improve the bioavailability of bioactive compounds [23]. Due to the structural particularities and the interaction between flavonoids, digestive enzymes and dietary components from the diet, the bioavailability of flavonoids in different stages of digestion, absorption and distribution is considered low [7]. In order to evaluate the effect of the coatings on the flavonoid behavior throughout the gastro-intestinal tract, an in vitro simulated study was undertaken, and results are discussed related to the structure–activity relationship at every stage during digestion. The results obtained for in vitro digestibility under simulated gastric (SGF) and intestinal fluids (SIF) show that the coating materials presented a protective effect on the flavonoids release. The sample presented an insignificant decrease (Figure 1) in flavonoids in gastric fluids with approximately 3% after 120 min of gastric conditions, meaning that almost all the bioactive compounds remain encapsulated.

On the other hand, the concentration of flavonoids increased with 15% in the intestinal tract, highlighting a release from the encapsulated matrix, followed by a decrease with approximatively 15% at the end of the reaction, suggesting that 85% of flavonoids are available after the entire digestion process. The results obtained in our study differ from those obtained by Sun et al. [24], who reported a significant decrease of 75% of the total anthocyanins during the simulated intestinal digestion. Kim et al. [25] suggested a significant decrease of 56% in simulated intestinal digestion model for cyanidin-3-O-glucoside in the mulberry extract, compared to that observed after simulated gastric digestion, whereas 69% cyanidin aglycone and 12% pelargonidin aglycone were recovered.

The obtained results confirmed that the selected matrix for the encapsulation of biologically active compounds extracted from yellow onion skins had a significant protective effect during digestion, allowing their controlled release.

### 3.4. Anti-Diabetic and Anti-Inflammatory Potential

The metabolic syndrome is a disorder of multiple etiologies characterized by disturbances of carbohydrate, chronic hyperglycemia and fat metabolisms [2]. The activity of the co-microencapsulated powder was assessed towards enzymes associated with metabolic syndrome, including α-amylase, pancreatic lipase and with pro-inflammatory enzymes, such as lipoxygenase, based on the reported evidences that phenolic compounds may freely interact with enzymes present in the digestive tract modulating their activity [26].

#### 3.4.1. α-Amylase Inhibitory Activity

According to the data from Global Health Estimates [27] worldwide, over 425 million people (20–79 years old) had diabetes, most of them with type 2 diabetes (352 million). In 2045, a number of 629 million people is expected to be diseased. Type 2 diabetes can be prevented by regular physical activity and a healthy diet. The inhibition of α-amylase is able to decrease hyperglycemia by suppressing the production or absorption of glucose from the gastrointestinal tract [28]. Several α-amylase inhibitors are very useful to treat diabetes, but they are expensive and have important clinical side effects. Medicinal plants have the potential to inhibit the saccharides hydrolyzing enzymes [12]. In other studies, Vasile et al. [6] obtained an inhibitory activity of 68% for an anthocyanin-based powder. Milea et al. [23] also reported an inhibition of 39% for a microencapsulated powder combining black rice and lavender extracts. In this study, the microencapsulated powder containing flavonoids extract was investigated for α-amylase inhibition. An inhibition ratio result of 76.40 ± 2.30% obtained in this study demonstrates that the sample may be considered for anti-diabetic potential.

#### 3.4.2. Lipase Inhibitory Activity

One of the most recommended treatments for weight management and obesity is based on the inhibition of pancreatic lipase, which is able to divide triacylglycerols into absorbable monoacylglycerol and fatty acids [2]. Natural antioxidants sources may be studied for the inhibition of lipase in order to substitute drugs. In this context, co-microencapsulated powder showed an inhibitory activity of 82.58 ± 3.36%. de la Garza et al. [29] also demonstrated a correlation between polyphenols from several dietary supplements and fruits and lipase inhibition.

#### 3.4.3. Lipoxygenase Inhibitory Activity

The effect of microencapsulated powder was tested against a pro-inflammatory enzyme, namely lipoxygenase. Products of this enzyme are important mediators of inflammation. Quercetin, kaempferol and its derivatives seem to be especially effective lipoxygenase inhibitors, while quercetin has inhibitory effect of PhosphoLipase A2 in human leukocytes [30]. The powder showed an inhibition rate of 49.01 ± 0.62%. Costamagna et al. [2] studied the inhibition effect of chanar fruit and obtain IC_50_ value of 48 ± 2 µg/mL. According to these results, it can be mentioned that the obtained functional ingredient can be valorized in order to achieve some desired beneficial health effects.

### 3.5. In Vitro Cytotoxicity of the Microencapsulated Powders

**Figure 2:**
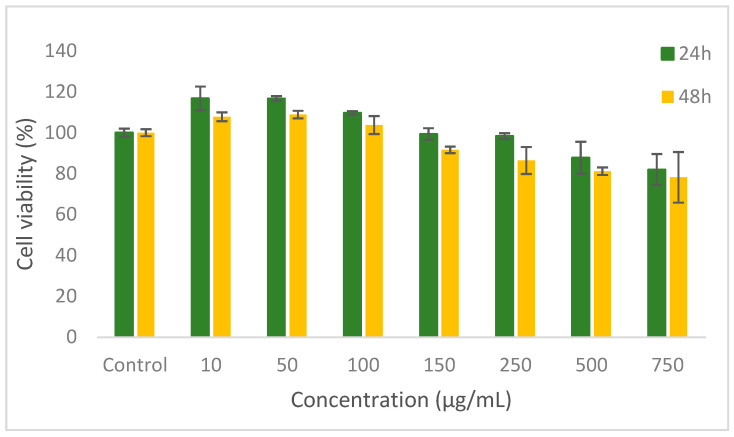
Cell viability of L929 fibroblasts cultivated in the presence of vegetal extracts, onion for 24 h and 48 h, respectively, determined by NR assay. The results were expressed as percentage relative to the control culture (untreated), considered 100% viable. The values represent mean ± SD (*n* = 3). *p* < 0.05, compared to control.

The in vitro cytotoxicity profile and cell viability were evaluated in L929 fibroblast cell culture by NR assay. The results showed that the co-microencapsulated compounds extracted from yellow onion skins did not show a cytotoxic effect up to the 500 μg/mL concentrations, after 24 h and up to the 250 μg/mL after 48 h of cultivation (>80% cell viability) (Figure 2).

The values of cell viability varied between 70.9–116.7% and decreased with increasing concentration. A moderate decrease in cell viability was registered for higher concentrations of 750–1000 μg/mL after 24 h and for 500–1000 μg/mL after 48 h treatment. These data illustrate the cytocompatibility of the powder up to 500 μg/mL. In addition, concentrations of powder in the range of 10–100 µg/mL stimulated the cell proliferation after 24 and 48 h of cultivation, compared to the control culture.

**Figure 3:**
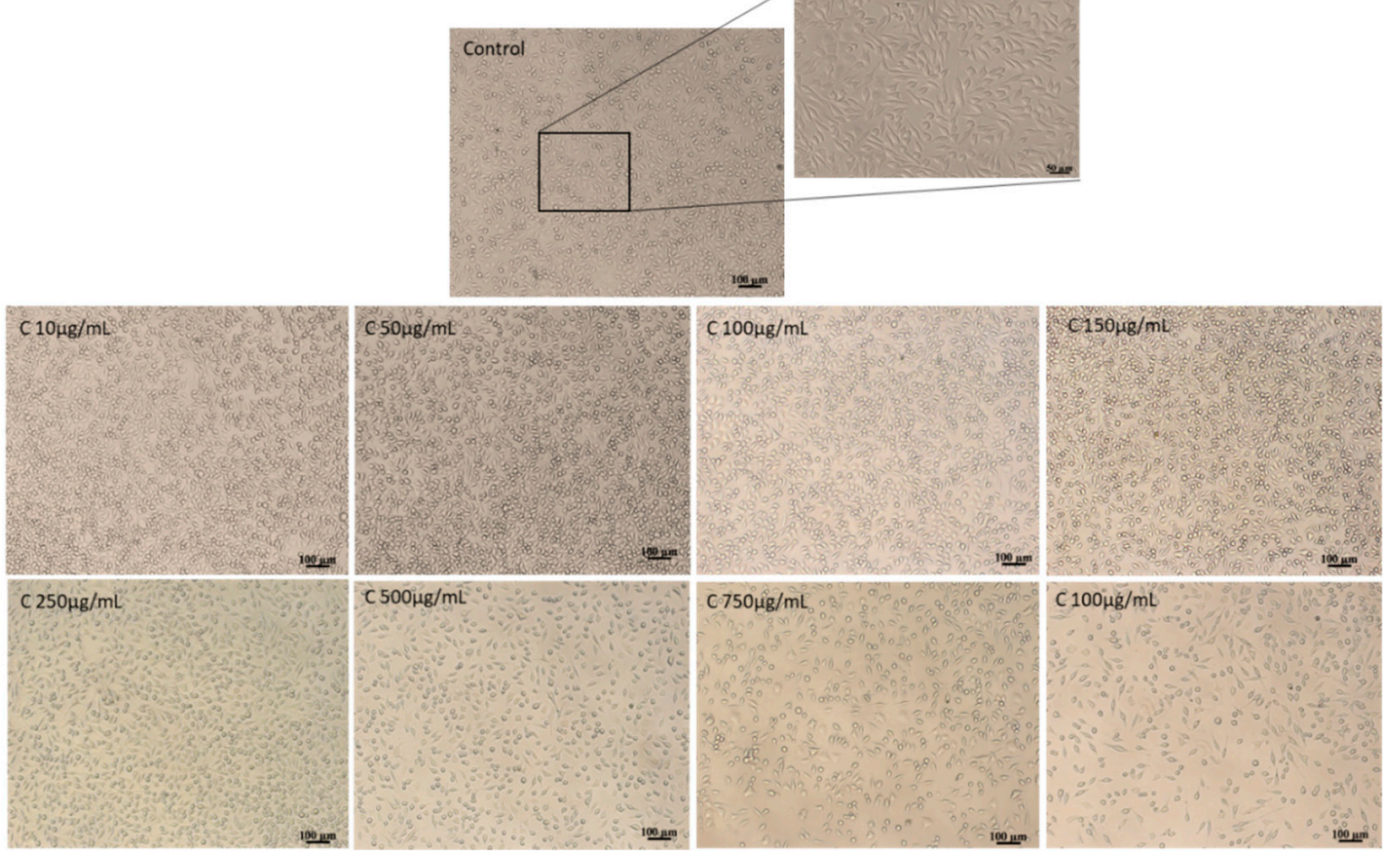
Light micrographs of L929 cells treated with onion extracts for 48 h.

Cell morphology observations were in accordance with NR quantitative data (Figure 3).

From the images, one can see that the cells treated with the co-microencapsulated powder are similar to the control culture cells, keeping their normal fusiform phenotype, characteristic for fibroblast cells. Additionally, a homogeneous distribution of cells on the culture plate is observed, with a density similar to the control culture. At concentrations of 10–100 µg/mL for powder, the cell density was higher when compared to the control culture. Higher concentrations >750 μg/mL of the vegetal extracts moderately decreased the cell density in treated cultures.

Our results are similar to Shi et al. [31], which showed that flavonoid-rich extracts of onion skins were not toxic in normal cell cultures, in the tested range of concentrations of 50–100 µg/mL. However, the results obtained in the present study indicate a significant increase in cytocompatibility, with cell viability higher than 80% for powder concentrations up to 500 µg/mL.

### 3.6. Characterization of New Formulated Food Product

In order to test its functionality, the powder was added into a selected matrix, in different ratios. Therefore, 2 variants of soft cheese were obtained: variant V1 (1%) and variant V2 (2%) and a blank without powder (C).

The obtained food products were analyzed in terms of bioactives stability and LAB viability for 21 days, during storage at 4–6 °C. As expected, the differences in bioactives and antioxidant activity between both samples correlates with the added quantity of powder (Table 2). When compared with a previous study from Milea et al. [23], it can be observed that in this context, the phytochemical content decreases with 43% for TFC, 35% for TPC and 8% for antioxidant activity, in case of variant V1 and with 47% for TFC, 31% and 9% for antioxidant activity in the case of variant V2 (Table 2).

The concentration decrease in bioactives and antioxidant activity could be supported by the increasing viability of LAB after 21 days. The microencapsulated powder stimulates the growth of *L. casei* 431. The combination between onion skins polyphenols, MD, I and WPI plays a prebiotic role for LAB. Likewise, Ma et al. [32] reported that the polyphenols found in green tea infusion stimulated the growth of *L. casei*. Otherwise, at the end of the storage, a significant number of antioxidant compounds remain in the final product, suggesting that it can be considered a functional food. This study demonstrates that the phenolic compounds have a synergic effect, supporting the LAB growth.

The cell viability of the co-microencapsulated LAB during storage at 4 °C, ranged from 6.66 to 7.51 log CFU/g DW after 21 days for samples V1 and V2. Therefore, for sample C, after 21 days, the viability of lactic acid bacteria decreased by 0.5 log CFU/g DW compared to samples V1 and V2 in which an increase in 1 log CFU/g DW was observed. As the minimum viability required for a product to be considered probiotic is 10^6^ CFU/mL [33], the addition of co-microencapsulated powder in fresh cheese increases the functionality of the product.

## 4. Conclusions

In recent years, people have become more interested in the development of a healthier lifestyle. The consumption of value-added products with the potential to prevent or manage the risk of specific illness have become popular. Yellow onion skins are an important source of naturally colored antioxidants, namely flavonoids. In this study, the flavonoids were valorized by extraction, co-microencapsulation with lactic acid bacteria and the development of a multifunctional ingredient. The encapsulation used a biopolymeric complex, involving the use of whey protein isolates, maltodextrin and inulin, by freeze-drying with the addition of *L. casei*. The resulting powder showed remarkable properties in terms of phytochemical profile, antioxidant, anti-diabetic, anti-inflammatory potential and also a significant cytocompatibility. High encapsulation efficiency was obtained for both flavonoids and lactic acid bacteria. The results obtained for in vitro digestibility showed that the coating materials presented a protective effect on the flavonoids release. Physical properties of the microencapsulated powder were investigated, and the result may be correlated with their stability on storage. In order to test the potential for food functionalization, the powder was added into a food model system (soft cheese cream). A stimulating effect on viability of *L. casei* was noticeable after 21 days in the variants with microencapsulated powder added. A synergic effect was observed between lactic bacteria and polyphenols in this study. These results confirmed that the encapsulated flavonoids from onion skins and lactic bacteria have the potential of being used to obtain functional foods or nutraceuticals.

## Figures and Tables

**Table 1:** The extract and powder phytochemical characterization.

Selected Phytochemical	Extract	Powder
Total flavonoid content (mg quercetin equivalents (QE)/g dry weight (DW))	229.14 ± 3.05 ^a^	89.49 ± 4.12 ^b^
Total polyphenol content (mg gallic acid equivalents (GAE)/g DW)	96.06 ± 2.70 ^a^	34.17 ± 1.79 ^b^
Antioxidant activity (mM Trolox/g DW)	101.19 ± 0.53 ^a^	39.27 ± 0.45 ^b^

Means that do not share a letter as a superscript (a, b) are significantly different.

**Table 2:** Storage stability of microencapsulated phenolic compounds in soft cheese.

Sample	Flavonoids, mg QE/g DW		Polyphenols, mg GAE/g DW		Antioxidant Activity, mM Trolox/g DW	
	0	21 Days	0	21 Days	0	21 Days
V1	4.81 ± 0.32 *	2.75 ± 0.23	4.68 ± 0.24	3.06 ± 0.24	1.95 ± 0.01	1.81 ± 0.01
V2	5.87 ± 0.22	3.16 ± 0.11	5.15 ± 0.29	3.60 ± 0.14	2.01 ± 0.01	1.83 ± 0.01

Values show average data of triplicate analyses, V1—soft cheese with 1% addition of microencapsulated powder, V2—soft cheese with 2% addition of microencapsulated powder; *—standard deviation.
